# Wearable Devices and New Frontiers in Smart Health Monitoring

**DOI:** 10.3390/s26072097

**Published:** 2026-03-27

**Authors:** Juan A. Holgado-Terriza, Pablo Pico-Valencia, Zilu Liang

**Affiliations:** 1 Software Engineering Department, Research Centre for Information and Communication Technologies (CITIC-UGR), University of Granada, 18071 Granada, Spain; pablo.pico@ugr.es; 2 Ubiquitous and Personal Computing Lab, Kyoto University of Advanced Science (KUAS), Kyoto 615-8577, Japan; liang.zilu@kuas.ac.jp

## 1. Introduction

The rapid evolution of technologies for remotely monitoring people’s health has driven the creation of a variety of innovative solutions with potential to automate, transform, and optimize traditional healthcare delivery [1]. In this context, wearable devices are some of the major innovations supporting a revolution in hospital services, allowing patients to be monitored outside hospital settings. Using data collected by integrated biosensors, it is possible to predict abnormal health events [2] and implement preventive treatment before critical conditions arise [3].

Currently, a wide variety of specialized wearable devices have been designed to monitor human health, including skin-, textile-, and biofluid-based wearables [4]. Some of these devices and/or biosensors are commercially available in the form of smart garments, smartwatches, foot and hand devices, sensor patches, e-tattoos, e-skins, or implants [5], whereas others remain in the experimental stage, undergoing clinical trials—such as the painless bilirubin-monitoring wearable for infants, which has only been tested in adults [6]. Depending on the health variables to be measured, wearable devices can integrate heterogeneous miniaturized sensors, including mechanical, physiological, and biochemical types. With these sensors, it is possible to measure parameters such as body temperature and heart rate [3], glucose levels using spectrometry [7], and even brain activity via electroencephalography (EEG) to determine a person’s fatigue level [8].

The use of wearable devices in healthcare is increasingly diversifying, with applications reported in the literature achieving high levels of accuracy in different real-world settings. Examples include physical activity monitoring systems for everyday users or professional athletes (e.g., vital signs and motion), personalized tracking of patients with chronic conditions (e.g., stroke and neurological diseases and respiratory disorders) [9], remote monitoring of older adults living alone (e.g., cardiovascular, musculoskeletal, and mental health conditions) [10], and detecting rare pathologies for which no effective diagnostic methods currently exist, such as fetal movement [11]. The development of these systems relies on the integration of wearable devices with artificial intelligence (AI), data science, and the Internet of Things (IoT). Furthermore, these systems are frequently combined with statistical models to evaluate the reliability and accuracy of the data and the recommendations derived from the biosensors embedded within these wearable systems.

In the healthcare field, the development of such innovations involves a complex and time-consuming process, from the initial concept to acceptance by the medical and scientific community. Clinical trials involving a substantial number of patients are required to ensure the generalizability of results, which makes wearable devices expensive to develop and validate. However, as costs continue to gradually decrease, the next generation of wearable devices for continuous health monitoring faces significant challenges in terms of interoperability, security, miniaturization, communication, and energy consumption.

Regarding the interoperability of wearable devices, compliance with industry standards for their manufacturing and use must be established and monitored [7]. In this regard, the materials used in the construction of biosensors must also be standardized to ensure that they are safe for and biocompatible with humans. A wide range of approved materials are currently employed to fabricate sensors—polydimethylsiloxane, polymethylmethacrylate, polycarbonate, polytetrafluoroethylene, polypropylene, polyimide, polyethylene terephthalate, glass, silicon, cellulose paper, epoxy resin, gold nanoparticles, graphene nanoplates, and acrylic adhesives, among others [12,13]. Many of these materials are chosen for their flexibility and adaptability to the human body, whereas others are selected for their electrical conductivity. However, in the coming years, a new generation of wearable devices is expected to emerge. These will be developed from existing devices and novel materials fabricated through 3D printing technologies. Likewise, the resolution of the current printing techniques used in biosensor manufacturing, such as gravure, flexography, inkjet, and screen printing, must be increased from the micrometer (µm) scale [14] toward nanoscale printing (<1 µm) or hybrid micro–nano fabrication. This transition will enhance the development of nano biosensors, establishing them as a fundamental component of the next generation of wearable technologies.

Along these lines, as technology continues to advance and chip manufacturing reaches nanometer scales, the ongoing miniaturization of biosensors—key components of wearable devices—will enable the next generation of wearables to become less intrusive, seamlessly integrated into everyday clothing, and even implanted into human skin. In the forthcoming decades, humans will be more connected than ever, to the point where wearables could not only be monitored remotely but even reprogrammed. This development poses significant risks, rendering humans more vulnerable to a range of threats. Research will also continue on new energy-harvesting mechanisms to extend the operational lifespan of wearable devices. The next generation of wearables will incorporate printed structures with nanoelectrodes, allowing low-current readout circuits suitable for energy-autonomous systems [14].

Furthermore, as the utilization of wearable technology becomes increasingly popular and widely adopted, the significance of implementing robust cybersecurity measures must be acknowledge, rather than perceiving them as a cosmetic concern. Because wearable devices rely on wireless communication and Internet connectivity, they are inherently vulnerable to unauthorized access. Such access could enable the execution of a cyber-attack on the human body, which has, to date, been predominantly observed in the context of digital networks, servers, and cloud systems. In this regard, future wearables will need to be compatible with new generations of wireless networks, such as 5G [15] and 6G, which are still under active investigation in several countries. These advancements will benefit future wearable devices by enabling smaller form factors, reduced weight, and improved energy efficiency. Earlier projections anticipated that the healthcare market would increasingly depend on 6G connectivity, with potential to further empower the Intelligent Internet of Medical Things (IIoMT) ecosystem [16].

The advent of 5G and 6G technologies will catalyze the evolution of wearable devices designed for health monitoring. Nevertheless, novel challenges will also arise in terms of cybersecurity, privacy, and health data governance, in conjunction with the development of innovative materials for the fabrication of biosensors at the micro- and nanoscale levels. Future generations of sensors will be capable of analyzing health variables derived from previously unexplored biological signals (e.g., sweat, tears, saliva, and blood). Likewise, a key future trend points toward the creation of multimodal wearables made from advanced and flexible materials. These devices are characterized by their minimal energy consumption, low invasiveness, and user-specific personalization [17].

This Special Issue highlights several aspects of the gap between the current generation of wearables and the upcoming next generation, achieved through advances in wireless communication and device miniaturization. The focus of this Special Issue extends beyond the proposal of new wearable devices: it also explores the optimization of sensors through the use of new materials, the application of AI for more accurate data analysis, and the use of wearables in medical training, enabling healthcare professionals to develop clinical practices with a reduced likelihood of human error. The studies presented in this Special Issue underscore the scientific community’s ongoing need to continue exploring the field of wearable health monitoring and to propose new ideas that challenge the current boundaries of biosensor technology. The intention of this editorial is not to delve deeply into each individual study but rather to encourage researchers to examine them and to build upon their findings—laying the groundwork for the next generation of wearable devices designed for continuous health monitoring.

## 2. An Overview of the Contributions

This Special Issue of *Sensors*, titled “Wearable Sensors for Continuous Health Monitoring and Analysis,” features fifteen selected manuscripts, comprising fourteen original research articles (1–14) and one comprehensive literature review (15). All manuscripts were subjected to a rigorous review process in accordance with the editorial policies and quality standards of the Multidisciplinary Digital Publishing Institute (MDPI). The published contributions are listed in the “List of Contributions” before the References.

This Special Issue reflects the international scope of the field of wearable sensors for the continuous monitoring of human physiological/biometric parameters. The researchers’ studies focused on accurately measuring and monitoring the following conditions: glucose levels, fetal movement, oxygen saturation, pressure, heart rate, bilirubin, hand movements, electroencephalographic activity, skin temperature, knee flexion, skin thermal properties, and general health parameters.

The published studies are representative of the work conducted by academic institutions located in a variety of countries, including the USA (6), Japan (2), Spain (2), China (1), Portugal (1), Greece (1), Latvia (1), and France (1). This demonstrates the global interest in continuous health monitoring across Europe, the Americas, and Asia. Primarily, the investigations involved experiments that mainly utilized wearable technology with IoT and predictive models to enable more precise, reliable tracking, tailored to each person’s condition and lifestyle. This has translated into a variety of methodological approaches, encompassing the design of new devices and sensory architectures, and the application of advanced techniques in the fields of AI, data science, biomedical engineering, and digital medicine. These approaches have yielded significant findings that describe the future of smart digital health that will involve the use of digital innovations to facilitate earlier diagnoses and interventions [18].

In order to perform a more specific analysis of each of the 14 published articles (excluding the review), Table 1 presents the studies based on six criteria, including clinical validation and accuracy, advanced signal processing and edge AI, energy efficiency and autonomy, multimodal sensor fusion, material innovation and form factor, and data integrity and security of health monitoring systems using wearable devices.

First and foremost, clinical validation against recognized medical gold standards remains a fundamental prerequisite for translating wearable technologies from experimental prototypes into routine clinical practice. Within this Special Issue, a subset of studies are distinguished by achieving a high level of compliance with this criterion. Articles such as 2, 6, 8, 9, and 14 explicitly benchmarked wearable-derived measurements against established clinical references, including ECG, EEG, continuous glucose monitoring (CGM) systems, or standardized thermal and bilirubin assessment methods. These studies provide robust statistical evidence through correlation analyses, error metrics, Bland–Altman plots, or clinically accepted evaluation grids (e.g., Clarke error grid), thereby strengthening their medical credibility. Complementing this, other studies in this Special Issue (4, 7, and 10–13) directed their focus toward technological feasibility and sensor innovation. These researchers relied on controlled experimental settings or annotated datasets to demonstrate proof-of-concept efficacy. Although these studies are crucial for pushing the boundaries of form factor and signal acquisition, the path toward widespread clinical adoption will require future iterations of this research to bridge the gap in rigorous validation against hospital-grade equipment, particularly for diagnostic and decision-support applications.

Equally important, advanced signal processing and intelligent data interpretation constitute the cognitive backbone of modern wearable systems, enabling the extraction of meaningful physiological information from noisy and artifact-prone signals. In this Special Issue, multiple studies addressed this challenge by leveraging machine learning (ML), Bayesian modeling, or personalized analytics to enhance robustness and interpretability (1, 3, 4, 6, 7, 9, 11 and 13). Notably, 9 and 11 stand out for their sophisticated treatment of uncertainty, artifact mitigation, and user-specific adaptation. Nevertheless, despite these algorithmic advances, a gap remains in deployment architectures: most processing pipelines are still executed offline or in the cloud, with limited explicit implementation of on-device or edge-based intelligence. Consequently, future research must increasingly focus on lightweight, explainable, and energy-aware algorithms capable of operating directly on wearable hardware. This shift is essential for reducing latency, ensuring data privacy, and enabling real-time clinical feedback required for acute care scenarios.

In parallel, energy efficiency and device autonomy remain among the most critical yet least systematically addressed challenges in continuous health monitoring. Only a small number of contributions in this Special Issue explicitly prioritized energy-aware design, either through the development of battery-less or passive sensing architectures (12) or through deliberately lightweight algorithms optimized for real-time execution with minimal computational overhead (13). By contrast, the majority of researchers implicitly assumed the availability of sufficient power without reporting detailed consumption metrics or optimization strategies. However, as wearables move toward long-term, continuous, and unsupervised deployment in real-world environments, energy autonomy will become a decisive factor determining usability. Therefore, future generations of wearable systems must increasingly incorporate ultra-low-power architectures, duty-cycling strategies, and energy-harvesting mechanisms to ensure sustainable operation over extended periods.

Moreover, multimodal sensor fusion emerges as a key enabler for overcoming the inherent limitations of single sensor approaches and achieving a more holistic understanding of human health. Several studies in this Special Issue exemplify the potential of this paradigm by integrating heterogeneous data streams, including physiological, contextual, and historical information (2, 3, 9 and 11). These contributions show that combining multiple sensing modalities for sensing, for example, heart rate, motion, temperature, behavioral data, or longitudinal records, substantially improves robustness, personalization, and contextual awareness. In contrast, although unimodal systems remain valuable for specific, targeted applications, they often struggle to maintain reliability in complex real-world scenarios where artifacts and environmental noise are prevalent. Consequently, multimodal sensing and data fusion are expected to become foundational components of future clinical-grade wearable systems.

At the hardware level, material innovation and form factor design play decisive roles in determining comfort, biocompatibility, and long-term user adherence. In this context, several contributions in this Special Issue advance flexible electronics, epidermal sensors, 3D-printed architectures, and novel functional materials (8, 10 and 14). These studies demonstrate that progress in wearable health monitoring is not driven solely by algorithms but also by innovations in materials science that directly influence signal quality, mechanical stability, and user experience. However, challenges regarding the mass manufacturability and washability of these novel interfaces remain to be fully addressed. Looking forward, the convergence of advanced materials, miniaturization, and hybrid micro–nano fabrication techniques will be pivotal in enabling wearables that are not only increasingly unobtrusive but also sufficiently durable and scalable for seamless integration into everyday life.

Finally, data integrity and security represent critical yet largely underexplored dimensions within experimental wearable research. Despite the sensitive nature of physiological and medical data, the explicit consideration of these factors in this Special Issue is largely confined to the review contribution (15), which discusses these aspects from a conceptual and regulatory perspective. In the experimental studies, technical implementations of security mechanisms—such as lightweight encryption, secure transmission, or mutual authentication protocols—were mostly absent. This gap is particularly concerning given the resource constraints of wearable devices and their reliance on wireless connectivity, which expands the attack surface. Moving forward, ensuring data security must become an integral part of wearable system design, using security-by-design approaches. Future systems must successfully balance privacy protection with the stringent constraint of computational efficiency and energy consumption. Ultimately, trust in wearable health technologies will depend on not only clinical accuracy but also their ability to safeguard user data in real-world deployments.

## 3. Conclusions

The articles compiled in this Special Issue address the topic of wearable devices for health monitoring from diverse perspectives. Although the initially expectation was that most, if not all, of the studies would propose new biosensors or wearable devices that would be coherently integrated through systems or applications to support the monitoring and generation of physiological data to assist physicians in diagnosing or making decisions regarding pathologies affecting individuals at home or patients in hospitals, the research requirements in this field extend far beyond that scope.

Regarding the biosensors and wearables presented in this Special Issue, the contributions achieve significant advancements toward the design of new architectures and the development of functional materials that expand the possibilities of continuous health monitoring. The studies propose the use of optical and thermal sensors, as well as flexible electromagnetic and pressure devices, with a focus on miniaturization, sensitivity, and adaptability to the human body’s taxonomy. These innovations can be observed in the preparation of an ionic photosensitive resin for pressure sensors (5), the development of an optical system for transcutaneous bilirubinometry (6), the validation of an in-ear EEG using dry electrodes (8), the design of an electromagnetic sensor for bilateral knee flexion monitoring (12), and the creation of a noninvasive skin calorimeter (14). Collectively, these studies reveal a trend toward the integration of advanced materials and 3D printing technologies, driving the development of more reliable, less invasive, and energy-autonomous biosensors that consolidate the technological foundation for the next generation of intelligent medical wearables.

In another line of research, a number of researchers have proposed methods and algorithms focused on analyzing the data generated by biosensors to enable more accurate diagnostics. These studies highlight the role of AI, ML, and statistical methods as essential tools for the interpretation and prediction of physiological parameters. Examples include a human activity recognition model using knowledge distillation (1), a continuous and noninvasive glucose prediction model based on multimodal data (2), the detection of fetal movement using abdominal IMUs (3), a multiscale model for sleep apnea classification (4), the BEGP statistical model for heart rate signal fusion (9), real-time fall detection algorithms (7 and 13), and a personalized model for well-being and empathy based on explainable AI (11). This research area is essential and closely aligned with the development of biosensors, as raw data alone are insufficient for informed decision-making. Therefore, the implementation of such methods guarantees the generation of reliable and actionable information and even contributes to the training of healthcare professionals (7) by reducing clinical errors and enhancing timely preventive care.

Finally, we emphasize that, collectively, these practical contributions help to consolidate a digital health ecosystem. In this ecosystem, wearable devices have the capacity to measure, learn, predict, and assist healthcare professionals. This development represents a significant step forward toward smarter, more personalized, less invasive, and preventive health monitoring. In this regard, researchers are encouraged to continue advancing in this field and to form interdisciplinary teams with the aim of developing innovative solutions that will enable the next generation of health-monitoring wearables to be a tangible reality. Furthermore, research initiatives that concentrate on intelligent health monitoring using wearable technology must be encouraged, particularly through reviews that provide a clear overview of the current progress and emerging trends. For instance, the authors of 15 examined existing driver health monitoring systems based on wearable sensors for the detection of drowsiness and stress while driving.

## Figures and Tables

**Table 1:** Uses of wearable devices and sensors for continuous health monitoring: 1 = clinical validation and accuracy; 2 = advanced signal processing and edge AI; 3 = energy efficiency and autonomy; 4 = multimodal sensor fusion; 5 = material innovation and form factor; 6 = data integrity and security.

Article	Device/System	Scope	1	2	3	4	5	6
1	Human Activity Recognition (HAR)	Demonstrated the effectiveness of image representation-driven knowledge distillation (KD) for improved interpretation of inertial sensor time-series data, such as those from accelerometers (which measure physical activity and movement), to classify different daily activities in a manner sufficiently light and efficient to run directly on the wearable device	—	X	X	—	—	—
2	Glucose Prediction (XR Model)	Proposed an approach that utilizes multimodal data from wearable sensors (heart rate, skin temperature, physical activity) for the development of continuous and noninvasive glucose prediction models (using XGBoost) based on biological sex and the free-living conditions under which the person lives	X	X	—	X	—	—
3	Fetal Movement Detection (Wearable IMU)	Developed machine learning models (CNN, BiLSTM, RF) using wearable sensors (inertial measurement units (IMUs)) placed on the mother’s abdomen for continuous and objective detection of fetal movement and its distinction from maternal movement	X	X	—	X	—	—
4	Multi-Scale SpO_2_ Apnea Detector	Proposed a multiscale feature engineering approach to develop machine learning models (naïve Bayes, CatBoost, XGBoost, MLP) for the detection and severity classification of sleep apnea using oxygen saturation signals (SpO_2_)	X	X	—	—	—	—
5	3D-Printed Ionic Pressure Sensor	Prepared an ion compound photosensitive resin for digital light processing (DLP) 3D printing to create flexible pressure sensors that exhibit high sensitivity and fast response time, opening possibilities for the structural design of wearable sensors adaptable to different parts of the human body	X	—	X	—	X	—
6	Continuous Transcutaneous Bilirubinometry System	Created an optical wearable device for the continuous and noninvasive monitoring of transcutaneous bilirubin (TcB) and address the prediction of device placement status (signal quality index) using machine learning (support vector machine) to ensure reliable estimates of bilirubin concentration across different light and dark skin tones	X	X		X	X	—
7	Enhanced 2D Hand Pose Estimation System	Trained a hand pose estimation model (ResNet) to track the movements of hands wearing medical gloves of different colors, with the aim of evaluating and training healthcare professionals to reduce clinical procedure errors	X	X	—	—	—	—
8	In-Ear EEG Wearable (Naox)	Demonstrated that in-ear electroencephalography (EEG) signal can offer recordings comparable to those of traditional EEG, with correlations exceeding 0.8 in most tasks, thus consolidating its viability as an alternative in the field of neurological wearables for portable and ambulatory brain monitoring using dry electrodes in the ear canal	X	X	X	—	X	—
9	BEGP (Basis-Expansion Gaussian Process) Model	Proposed the basis of a new model called the Basis-Expansion-Based Gaussian Process (BEGP) that merges historical data with new records to correct errors and improve data quality, estimate individual heart rate patterns in older adults, and thus provide personalized care and prevent adverse events	X	X	—	X	—	—
10	Thermal Plantar Orthotics Study	Evaluated the thermal response of three lining materials for plantar orthoses (polyethylene (PE), ethylene-vinyl acetate (EVA), and PE-EVA copolymer) to determine which material best controls the temperature increase during continuous use, proposing guidelines for the design of new wearables that prevent overheating	X	—	—	—	X	—
11	Personalized ML for Wellbeing and Empathy	Implemented personalized machine learning (PML) under an N-of-1 approach, where each participant had their own predictive model. This model was trained using physiological features (heart rate, step count, speed, duration, calories burned, and exercise performed) and behavioral features (mood, sleep hours, and screen time) to obtain more accurate and individually adaptable predictions of well-being and increase empathy in healthcare professionals	X	X	—	X	—	—
12	Wearable Loop Sensor for Bilateral Knee Flexion	Proposed a wearable loop sensor to monitor bilateral human knee flexion and conducted a detailed analysis of error sources to optimize the accuracy of human joint movement measurement	X	—	X	—	—	—
13	PIPTO Fall Detection Pipeline	Introduced a heuristic-based algorithm (PIPTO; *píptw*, fall) for accurate, real-time fall detection, relying exclusively on three-axis accelerometers integrated into wearable devices and using only the magnitude of the resulting acceleration vector	X	X	X	—	—	—
14	Skin Calorimeter	Validated a cutaneous calorimeter to monitor thermoregulation based on three thermal skin properties (heat flow, heat capacity and thermal resistance) and assist in the diagnosis of skin disorders in people	X	—	—	—	X	—
